# Myeloid cell iron uptake pathways and paramagnetic rim formation in multiple sclerosis

**DOI:** 10.1007/s00401-023-02627-4

**Published:** 2023-09-16

**Authors:** Annika Hofmann, Nik Krajnc, Assunta Dal-Bianco, Christian J. Riedl, Tobias Zrzavy, Celia Lerma-Martin, Gregor Kasprian, Claudia E. Weber, Francesco Pezzini, Fritz Leutmezer, Paulus Rommer, Gabriel Bsteh, Michael Platten, Achim Gass, Thomas Berger, Philipp Eisele, Roberta Magliozzi, Lucas Schirmer, Simon Hametner

**Affiliations:** 1 Department of Neurology, Medical Faculty Mannheim, Heidelberg University, Mannheim, Germany; 2 Division of Neuropathology and Neurochemistry, Department of Neurology, Medical University of Vienna, Vienna, Austria; 3 Comprehensive Center for Clinical Neurosciences and Mental Health, Medical University of Vienna, Vienna, Austria; 4 Faculty of Medicine, University of Ljubljana, Ljubljana, Slovenia; 5 Department of Neurology, Medical University of Vienna, Vienna, Austria; 6 Division of Neuroradiology and Musculoskeletal Radiology, Department of Biomedical Imaging and Image-Guided Therapy, Medical University of Vienna, Vienna, Austria; 7 Department of Surgery, Dentistry, Paediatrics and Gynaecology, University of Verona, Verona, Italy; 8 Mannheim Center for Translational Neuroscience, Medical Faculty Mannheim, Heidelberg University, Mannheim, Germany; 9 Mannheim Institute for Innate Immunity, Medical Faculty Mannheim, Heidelberg University, Mannheim, Germany; 10 Interdisciplinary Center for Neurosciences, Heidelberg University, Heidelberg, Germany; 11 DKTK Clinical Cooperation Unit Neuroimmunology and Brain Tumor Immunology, German Cancer Research Center, INF 280, Heidelberg, Germany; 12 Department of Neurosciences, Biomedicine and Movement Sciences, University of Verona, Verona, Italy

**Keywords:** Multiple sclerosis, Postmortem, Magnetic resonance imaging, White matter, Iron metabolism, CD163, Haptoglobin

## Abstract

In multiple sclerosis (MS), sustained inflammatory activity can be visualized by iron-sensitive magnetic resonance imaging (MRI) at the edges of chronic lesions. These paramagnetic rim lesions (PRLs) are associated with clinical worsening, although the cell type-specific and molecular pathways of iron uptake and metabolism are not well known. We studied two postmortem cohorts: an exploratory formalin-fixed paraffin-embedded (FFPE) tissue cohort of 18 controls and 24 MS cases and a confirmatory snap-frozen cohort of 6 controls and 14 MS cases. Besides myelin and non-heme iron imaging, the haptoglobin-hemoglobin scavenger receptor CD163, the iron-metabolizing markers HMOX1 and HAMP as well as immune-related markers P2RY12, CD68, C1QA and IL10 were visualized in myeloid cell (MC) subtypes at RNA and protein levels across different MS lesion areas. In addition, we studied PRLs in vivo in a cohort of 98 people with MS (pwMS) via iron-sensitive 3 T MRI and haptoglobin genotyping by PCR. CSF samples were available from 38 pwMS for soluble CD163 (sCD163) protein level measurements by ELISA. In postmortem tissues, we observed that iron uptake was linked to rim-associated *C1QA*-expressing MC subtypes, characterized by upregulation of *CD163*, *HMOX1*, *HAMP* and, conversely, downregulation of *P2RY12*. We found that pwMS with $\ge$ 4 PRLs had higher sCD163 levels in the CSF than pwMS with $\le$ 3 PRLs with sCD163 correlating with the number of PRLs. The number of PRLs was associated with clinical worsening but not with age, sex or haptoglobin genotype of pwMS. However, pwMS with *Hp2-1/Hp2-2* haplotypes had higher clinical disability scores than pwMS with *Hp1-1*. In summary, we observed upregulation of the CD163-HMOX1-HAMP axis in MC subtypes at chronic active lesion rims, suggesting haptoglobin-bound hemoglobin but not transferrin-bound iron as a critical source for MC-associated iron uptake in MS. The correlation of CSF-associated sCD163 with PRL counts in MS highlights the relevance of CD163-mediated iron uptake via haptoglobin-bound hemoglobin. Also, while *Hp* haplotypes had no noticeable influence on PRL counts, pwMS carriers of a *Hp2* allele might have a higher risk to experience clinical worsening.

**Supplementary Information:**

The online version contains supplementary material available at 10.1007/s00401-023-02627-4.

## Introduction

Multiple sclerosis (MS) is a prototypic chronic-inflammatory disease of the central nervous system (CNS), resulting in the formation of demyelinating lesions and progressive neurodegeneration [21]. Sustained smoldering demyelinating activity, which is beyond the stage of gadolinium enhancement of active MS lesions [2, 4], is accompanied by iron accumulation at lesion rims in a considerable proportion of chronic active lesions [12, 49]. Iron rims can be visualized by iron-sensitive magnetic resonance imaging (MRI), forming paramagnetic rim lesions (PRLs) [5]. PRLs have been associated with a more severe disease course [3], higher brain and spinal cord atrophy rates [14, 61, 63], increased serum neurofilament light chain (sNfL) levels and lower retinal layer thickness [29] in people with MS (pwMS) [15, 35], reflecting continuous neuro-axonal damage [13, 27]. PRLs slowly expand over time [12, 62], while lesion rims become attenuated over years of lesion persistence [1, 13, 62]. It is known that iron rims are linked to iron uptake in phagocytic myeloid cell (MC) subtypes, namely microglia and macrophages, at the edges of chronic active lesions [5, 24, 39, 48]. These MC subtypes have a pro-inflammatory phenotype [1, 12, 23], however, the precise cell type-specific iron uptake mechanisms are not well studied, and the main iron source for iron rim formation remains to be clarified.

In subcortical white matter (WM) areas of controls, the MC-specific haptoglobin-hemoglobin complex receptor CD163 [34] is mainly expressed on perivascular macrophages [64] and selectively upregulated on a subtype of MCs in active and chronic active MS lesion rim areas [60, 64, 65]. Notably, CD163 upregulation on iron-laden peripheral tissue macrophages has been linked to their pro-inflammatory activation status [55]. The extracellular domain of membrane-bound CD163 can be shed by TACE/ADAM17, forming soluble CD163 (sCD163) [43], and elevation of sCD163 in the cerebrospinal fluid (CSF) of pwMS has been associated with higher cortical lesion load, a marker of a more severe disease course [36]. The ligands for CD163 are iron-containing hemoglobin-haptoglobin complexes [34]. Haptoglobin (Hp) is an abundant plasma protein that binds iron-containing hemoglobin, liberated from, for example, intravascular hemolysis, thereby preventing oxidative stress. Three major human Hp haplotypes exist, *Hp1-1*, *Hp2-2* and *Hp2-1* [56], the frequencies of which show profound geographical variation [10]. Hemoglobin binding capacity is lowest among *Hp2-2* subjects due to its lower concentrations and a lower ability to bind hemoglobin [44]. The aim of this study was to elucidate the cell type-specific mechanisms and sources of iron uptake in MC subtypes at chronic active lesion rims with an emphasis on CD163 and its role in PRL formation in pwMS.

## Methods

### Postmortem tissue samples

Cohort A was used to explore iron and important iron-related proteins in MCs across different activity stages of MS lesions (Supplementary Table 1). It consists of formalin-fixed, paraffin-embedded (FFPE) autopsy brain tissue samples from 18 controls and 24 pwMS, approved by the ethics committee of the Medical University Vienna (535/2004/2016). Confirmatory cohort B consists of 23 snap-frozen tissue samples obtained from 6 controls (6 samples) and 14 pwMS (17 samples), provided by the UK Multiple Sclerosis Tissue Bank at Imperial college in London, following ethical approval by the National Research Ethics Committee in the UK (18/WA/0238). Controls displayed absence of neurological disease in the medical history and no brain lesions at neuropathological examination. For cohort B, only MS cases with chronic active lesions were included. Further epidemiological, clinical and basic pathological information for both samples is provided in Supplementary Table 1. Primary antibodies and antigen retrieval methods are listed in Supplementary Table 2.

### Histopathological techniques

For cohort A, 3 µm thick FFPE tissue sections were stained with hematoxylin & eosin (H&E) and Luxol fast blue–periodic acid Schiff (LFB-PAS) myelin staining. MS lesion staging was performed based on LFB-PAS stainings and immunohistochemistry (IHC) for the myelin proteolipid protein (PLP; n = 28; see Supplementary Table 2). For detection of iron, diaminobenzidine (DAB)-enhanced Turnbull blue (TBB) staining was applied as described [38]. IHC using DAB as chromogen was performed as described [6]. Iron, transferrin receptor (TfR, encoded by *TFRC*), scavenger receptor class A member 5 (Scara5, encoded by *SCARA5*), divalent metal transporter (DMT1, encoded by *SLC11A2*), natural resistance-associated macrophage protein 1 (NRAMP1, encoded by *SLC11A1*), CD163 (encoded by *CD163*), ferroportin (encoded by *SLC40A1*), hephaestin (encoded by *HEPH*) and hepcidin (encoded by *HAMP*) immunoreactivities were assessed on consecutive tissue sections. For cohort B, 16 µm thick snap-frozen tissue sections were stained for LFB-PAS and DAB-enhanced TBB staining for ferric and ferrous non-heme iron. For IHC, antibodies against MOG, CD68 and CD163 were applied (see Supplementary Table 2). MOG and CD68 IHCs were performed as described previously [53]. CD163 IHC on frozen sections was done on a DAKO Link 48 autostainer with one hour incubation at room temperature (RT).

### Fluorescence duplex and multiplex RNA in situ hybridization

For duplex and multiplex single molecule RNA in situ hybridization (ISH) performed on cohort B, the ACD bio-techne protocols for the RNAscope 2.5 HD Duplex [53] and Fluorescent Multiplex V2 assays were applied as previously described [53, 59]. The following human ACD bio-techne RNAscope assay probes (cat. no.) were used: *C1QA* (485451), *CD163* (417061), *HAMP* (411911), *HMOX1* (319851), *IL10* (602051), *P2RY12* (450391). In addition, human ACD bio-techne 3-plex negative (320871) and positive ISH probes (320861) were run in parallel as a quality control.

### Lesion type characterization

In cohort A, lesions were staged according to myelin degradation products in lysosomes of macrophages based on LFB-PAS histology and PLP IHC and the presence of activated CD68^+^ MCs. The following regions of interest were investigated: normal white matter (WM) of controls (CTRL), MS normal-appearing WM (NAWM) in at least 10,000 µm distance to any discernible lesion rim (LR) [21], WM surrounding lesions (peri-plaque WM, PPWM), early active areas (LC-EA) and late active areas (LC-LA) of active lesions (A) (n = 12, see Supplementary Table 1). For chronic active (CA) lesions (n = 11), we investigated PPWM, LRs, and inactive lesion cores (LC-I). Active lesions were identified by a hypercellular lesion core with a high density of MCs containing myelin degradation products. In active lesions, LR areas—although not as distinct as in chronic active lesions—were characterized by a border zone with a relative loss of myelin extending into the LC associated with the presence of phagocytosing MCs. Chronic active lesions showed a hypocellular, demyelinated lesion center but distinct rim formation by CD68^+^ cells. The inactive lesion (consisting of one NAWM, one PPWM and one LC-I) was characterized by a fully demyelinated, hypocellular lesion center and low presence of CD68^+^ cells throughout lesion areas. The identification of MCs was based on morphology. Cells were quantified with an Olympus BX50 microscope using a 40 × objective. 9 fields per ROI were counted using a grid covering 0.24 × 0.24 mm resulting in a total area of 0.52 mm^2^ per ROI. One lesion per case and one ROI per lesion and ROI type were evaluated for the data presented (Fig. 2). For cohort B, chronic activity of lesions was confirmed based on LFB-PAS and MOG IHC for few rim-associated myelin degradation products and CD68 IHC for the presence of MCs.

### Microscopy and image acquisition

For cohort B, images for quantification were taken using a Leica DMi 8 microscope equipped with a Leica DFC 7000 GT camera. Focus points were set at 20 × magnification at the area of interest, pictures were then imaged and exported as LIF files. Confocal images were taken using Leica TCS SP8, Nikon AX R or Nikon A1 microscopes. All confocal pictures were taken as z-stack images consisting of 10 to 20 layers with a 0.5 to 0.7-μm step size. Heights for z-stacks were identified manually by imaging DAPI.

### RNA marker quantification

To detect gene expression changes in chronic active lesion areas of cohort B, we defined 4 ROIs that were examined in every lesion. As described before [24], the following ROIs were examined: control WM (CTRL), MS NAWM in at least 10,000 µm distance from the lesion, MS PPWM in 100–400 µm distance to the LR, MS LC in 500–1000 µm distance to the LR. For the quantification of each sample, we took 4 images within 4 different ROIs (LC, LR, PPWM, NAWM) using a 20 × objective. Each image covers 0.05–0.1 mm^2^ depending on the lesion size and resulting in a total of 16 images per lesion. For controls, 4 CTRL ROIs per sample, each covering an area of 0.05–0.1 mm^2^, were quantified accordingly. Quantification analysis was performed manually using FIJI ImageJ (version 2.0.0). For RNA in situ hybridization assays, cells presenting two or more signals were considered as positive cells.

### MR imaging study

In the cross-sectional, double center retrospective MRI study part, 86 pwMS from the Department of Neurology of the Medical University of Vienna and 12 pwMS from the University Medical Centre Mannheim were included, yielding a pooled cohort of 98 pwMS. Clinical and demographic characteristics of the cohort are given in Table 1. Diagnosis of clinically definite MS was established according to the McDonald criteria at the time of diagnosis between Jan 1st, 1991 and Dec 31st, 2020. All pwMS met the following inclusion criteria: age ≥ 18 years, availability of a T1, FLAIR and SWI-based brain MRI scan at 3 T and a blood sample drawn at any time independent of the MRI. The study was approved by the ethics committees of the Medical University of Vienna (EK 1599/2021) and Mannheim (2017-830R-MA). MRI analysis and processing of blood samples for *Hp* genotyping were performed at the Medical University of Vienna.

**Table 1:** Demographics of the MR imaging study cohort

	Study cohort (n = 98)	0 PRLs (n = 37)	1–3 PRLs (n = 36)	$\ge$ 4 PRLs (n = 25)	p-value
Female^a^	57 (58.2)	27 (73.0)	16 (44.4)	14 (56.0)	**0.046**
Age (years)^b^	38.0 (10.5)	35.3 (9.0)	38.1 (10.8)	41.6 (11.4)	0.068
Disease duration (years)^c^	6 (2.8–11.3)	4 (2.5–7.5)	7 (1–12)	9 (5–15.5)	**0.013**
EDSS^c^	2.0 (1.0–3.5)	1.0 (0–2.8)	2.0 (1.0–3.2)	3.5 (2.1–4.4)	**0.001**
MSSS^c^	3.10 (0.94–5.87)	2.34 (0.45–5.35)	3.14 (1.24–5.87)	4.28 (2.49–6.52)	**0.034**
RMS patients^a^	76 (77.6)	33 (89.2)	28 (77.8)	15 (60.0)	**0.026**
CSF sCD163 (ng/ml)^c,†^	28.3 (22.9–39.2)	25.8 (23.0–29.7)	26.7 (12.6–37.9)	39.5 (29.9–56.5)	**0.035**
DMT^a^					
No DMT	18 (18.4)	5 (13.5)	10 (27.8)	3 (12.0)	0.144
Moderately effective DMT	36 (36.7)	18 (48.7)	11 (30.5)	7 (28.0)	
Interferon beta preparation	5 (5.1)	4 (10.8)	1 (2.8)	0 (0.0)	
Glatiramer acetate	11 (11.2)	4 (10.8)	5 (13.9)	2 (8.0)	
Dimethyl fumarate	18 (18.4)	10 (27.0)	4 (11.1)	4 (16.0)	
Teriflunomide	2 (2.0)	0 (0.0)	1 (2.8)	1 (4.0)	
Highly effective DMT	44 (44.9)	14 (37.8)	15 (41.7)	15 (60.0)	
Fingolimod	16 (16.3)	4 (10.8)	8 (22.2)	4 (16.0)	
Cladribine	3 (3.1)	0 (0.0)	0 (0.0)	3 (12.0)	
Alemtuzumab	6 (6.1)	2 (5.4)	1 (2.8)	3 (12.0)	
Natalizumab	5 (5.1)	2 (5.4)	1 (2.8)	2 (8.0)	
Anti-CD20 mAbs	14 (14.3)	6 (16.2)	5 (13.9)	3 (12.0)	
MRI characteristics^c^					
Number of T2-lesions	19 (10–35.3)	14 (8–33)	14.5 (8.3–24)	33 (24–50)	** < 0.001**
Number of PRLs	1 (0–4)	n.a	2 (1–2)	9 (5–14.5)	n.a
Total brain volume (ml)	1092.0 (1006.8–1165.8)	1093.7 (1015.9–1192.1)	1106.3 (1000.7–1214.5)	1072.2 (983.5–1129.2)	0.491
Total GM volume (ml)	669.6 (602.1–716.6)	673.4 (601.2–708.1)	666.6 (608.8–742.9)	667.6 (600.8–700.9)	0.771
Total GM cortical volume (ml)	518.1 (472.3–557.0)	521.3 (475.4–541.5)	515.3 (472.7–586.9)	506.6 (456.8–547.7)	0.844
Total WM volume (ml)	424.0 (388.3–474.0)	431.1 (410.5–465.3)	431.7 (374.2–492.6)	421.5 (370.2–462.8)	0.510
WM abnormalities volume (ml)	1.1 (0.4–3.6)	0.9 (0.2–3.5)	0.8 (0.1–3.6)	2.5 (0.7–5.9)	0.105
CSF volume (ml)	336.9 (286.0–381.8)	329.9 (272.5–363.6)	330.4 (289.5–380.3)	379.3 (288.4–404.3)	0.134
Thalamus volume (ml)	13.3 (12.1–14.2)	13.8 (12.7–14.1)	13.7 (12.5–14.7)	12.5 (11.5–13.6)	0.054
Haptoglobin genotype^a^					
Hp1-1	8 (8.2)	4 (10.8)	2 (5.6)	2 (8.0)	0.714
Hp2-2 or Hp2-1	90 (91.8)	33 (89.2)	34 (94.4)	23 (92.0)	

Numbers in boldface highlight significant p-values

^a^Number (percentage), ^b^Mean and standard deviation, ^c^Median and interquartile range

^**†**^Data available in 38 pwMS

*Anti-CD20* *mAbs* monoclonal antibodies against cluster of differentiation 20 (ocrelizumab, rituximab), *CSF* cerebrospinal fluid, *DMT* disease-modifying therapy, *EDSS* expanded disability status scale, *GM* grey matter, *MSSS* multiple sclerosis severity scale, *PRL* paramagnetic rim lesion, *RMS* relapsing multiple sclerosis, *sCD163* soluble cluster of differentiation 163, *WM* white matter

### MR imaging acquisition

In Vienna, all brain scans were performed on a Siemens Magnetom 3 T Trio (2015–2017) and Vida (2018–2020) MRI system, using a 32-channel radio frequency (RF) coil. Images were acquired between Jan 1st, 2015, and Dec 31st, 2020: isovoxel 1 mm^3^ 3D fluid-attenuated inversion recovery (FLAIR) (echo time TE = 398 ms, repetition time TR = 5000 ms, inversion time TI = 1800 ms, field of view (FOV) = 240 mm^2^, resolution = 0.9 × 0.9 × 0.9 mm^3^), 3D T1-weighted images (TE = 2.92 ms, TR = 1800 ms, TI = 900 ms, FOV = 256 mm^2^, resolution = 1 × 1 × 1 mm^3^) and multi-echo susceptibility-weighted image sequence (SWI) (TE 1 = 7.67 ms, TE 2 = 24.60 ms, TR = 35 ms, FOV = 220 mm^2^, resolution = 0.3 × 0.3 × 1.5 mm^3^) were acquired consecutively. In Mannheim, all MRI scans were performed on a Siemens Magnetom 3 T Skyra System, using a 20-channel head coil. The following sequences were used: 3D FLAIR (TE = 398 ms, TR = 5000 ms, TI = 1800 ms, FOV = 240 mm^2^, resolution = 0.5 × 0.5 × 0.9 mm^3^), 3D MPRAGE (TE = 2.49 ms, TR = 1900 ms, TI = 900 ms, FOV = 240 mm^2^, resolution = 0.9 × 0.9 × 0.9 mm^3^),, and SWI (TE = 20 ms, TR = 27 ms, FOV = 220 mm^2^, resolution 0.9 × 0.9 × 1.5 mm^3^).

### Evaluation of lesions in MR imaging

All supratentorial lesions of the periventricular, juxtacortical and deep WM in the frontal, parietal, temporal and occipital lobes [19] as well as infratentorial lesions of the cerebellum were analyzed by two independent raters (ADB, NK) with a long-standing expertise in MS imaging analysis. PRLs were defined as FLAIR-hyperintense lesions that were partially or completely surrounded by a pronounced and distinct SWI-hypointense rim. The presence of a central vein was not considered for rim evaluation. After both raters had made their decision, unclear lesions were discussed and an agreement was reached. The inter-rater agreement before matching was 98.7%. Volume of FLAIR and T1 lesion, and total brain volume were automatically assessed using the MorphoBox prototype imaging software normalized for age from Siemens Healthineers [54].

### DNA isolation for *Hp* genotyping

Blood was drawn from the patients into EDTA tubes and immediately processed by centrifugation for 15 min at 15,000 g at RT. Next, plasma was removed, and remaining blood cells were stored at − 80 °C. For DNA isolation, the Qiagen DNeasy blood and tissue kit together with the corresponding protocol for non-nucleated blood cells was used. All centrifugation steps were carried out at RT. Blood samples were removed from the freezer and instantly incubated in the water bath (37 °C) until the blood was liquified. For sample lysation and RNA subtraction, 100 µl of blood was mixed with 100 µl 1 × PBS, 20 µl Proteinase K (Qiagen) and 4 µl RNAse A (Qiagen) and incubated for 2 min at RT. After adding 200 µl Buffer AL (Qiagen) and incubation for 10 min at 56 °C on a rocking platform, 200 µl ethanol 100% was added and the mixture was transferred into the provided DNeasy mini spin column, placed in a 2 ml tube, and centrifuged at 8000 rpm for 1 min. The DNeasy mini spin column was transferred into a new 2 ml tube container, and enzyme inhibitors were removed by the following two washing steps: 500 µl Buffer AW1 (Qiagen) centrifuged at 8,000 rpm for 1 min followed by 500 µl Buffer AW2 and again centrifuged at 14,000 rpm for 3 min. DNA elution of the membrane was performed by placing the spin column into a new 2 ml tube, adding 100 µl RNAse free water, incubated for 1 min at RT and then centrifuged for 1 min at 8000 rpm. To increase DNA yield, this procedure was repeated. DNA yields (range of 3–90 ng/µl) were measured using the Qubit RNA/DNA work station (Thermo Fisher) to ensure a sufficient concentration of extracted DNA.

### *Hp* genotyping

*Hp* haplotypes were determined by allelic amplification in two distinct PCR reactions and separated by agarose gel electrophoresis using primers A (5 -GAGGG GAGCTTGCCTTTCCATTG-3) and B (5–GAGATTTTTGAGCCCTGGCTGGT-3) as well as primers C (5 -CCTGCCTCGTATTAACTGCACCAT-3) and D (5 -CCGAG TGCTCCACATAGCCATGT-3) according to Koch et al. [26]. All experiments were performed in duplicates.

### CSF sCD163 analysis

CSF samples were obtained at the time of diagnosis with a median time of ± 4 months from the MRI, according to Consensus Guidelines for CSF and Blood Biobanking [58]. After centrifugation, CSF supernatant and cell pellet were stored separately at − 80 °C. CSF analysis was optimized and performed by two independent investigators, blinded for the patients’ clinical and MRI features. The concentration of sCD163 (ng/mL/mgProt) was assessed by using the ultrasensitive Ella-Simple Plex technology (ELLA microfluidic analyzer, Protein Simple, Bio-techne), an automated ELISA, according to the manufacturer’s protocol. To verify the reproducibility and consistency of the results, all samples were run in triplicates.

## Statistical analysis

For *postmortem* data acquisition, each MS lesion was quantified individually. Regarding cohort A, one lesion per case was analyzed. For cohort B, some cases contributed with more than one lesion per case (see Supplementary Table 1), therefore, average values in the 5 different ROIs were used with each individual being represented by one mean value per lesion and ROI type. Statistical analysis was performed using GraphPad Prism 9 software. For cohort A and B, normality and log-normality tests were performed (Anderson–Darling, D’Agostino and Pearson, Shapiro–Wilk and Kolmogorov–Smirnov). If gaussian normal distribution was not assumed, non-parametric Kruskal–Wallis and Dunn’s multiple comparisons tests were performed to analyze expression of markers in 5 different regions (LC, LR, PPWM, NAWM, CTRL). If data showed an approximate gaussian normal distribution, indicated by p > 0.05 in the normality and log-normality tests, one-way ANOVA tests with Tukey´s multiple comparison tests were performed. For staining combinations lacking control tissue, paired analyses of the 4 regions (LC, LR, PPWM, NAWM) were performed using parametric and non-parametric tests.

Clinical data were analyzed using IBM SPSS Statistics for Windows (version 26.0). Categorical variables were expressed in frequencies and percentages, continuous variables as means and standard deviations (SD) or medians and interquartile ranges (IQR) as appropriate. Continuous variables were tested for normal distribution by the Kolmogorov–Smirnov test with Lilliefors correction. Mann–Whitney U-tests or Kruskal–Wallis tests were used to compare groups as appropriate. Box plots and scatter plots were used for graphical representation. Because of skewed data, Spearman’s Rank Correlation Coefficient was used to evaluate bivariate correlations between number of PRL and age, disease duration, EDSS and MSSS. In a multivariate linear regression model, the number of PRLs was used as a dependent variable, and age, sex, disease duration, EDSS and *Hp* genotype as independent variables. To explore the potential risks for the presence of PRLs, a multivariate binary logistic regression model was conducted with the presence of PRLs as dependent variable, and the variables mentioned above as independent variables. For further analyses, patients were grouped according to disease duration in early (< 5 years), middle (6–10 years), and late relapsing MS (RMS) phase (> 11 years). A sensitivity analysis was conducted by removing patients from the Mannheim cohort, in order to investigate a potential confounding influence of pooled datasets. Significance level was set at a two-sided p-value < 0.05 with Bonferroni correction for multiple testing. We reported statistically significant *p*-values as follows: *p < 0.05, **p < 0.01, ***p < 0.001.

## Results

### Enhanced iron uptake and expression of iron-related proteins in MC subtypes

**Fig. 1:**
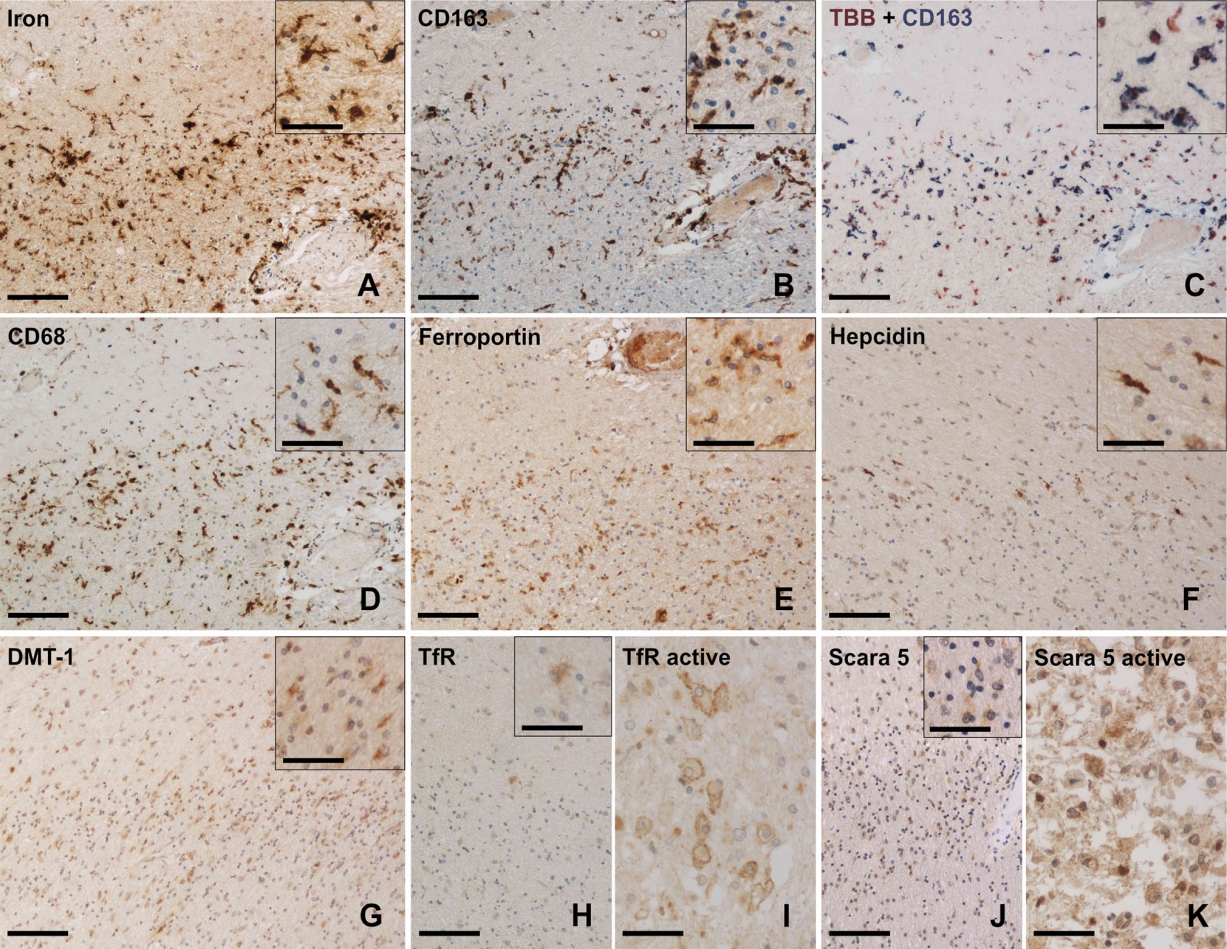
Representative images of iron (**A**) and iron-related proteins (**B–K**) at a rim of a chronic active MS lesion. **A** Selective iron accumulation in MCs at the demyelinating lesion rim and around blood vessel (right corner); note some iron-positive microglia display dystrophic morphology (inset). **B** The haptoglobin-hemoglobin receptor CD163 is strongly enriched at lesion rims and (**C**) present on iron-laden MCs based on morphology. **D** Activated rim MCs show a strong immunoreactivity for the phagocytosis marker CD68. **E** Lesion rim-associated MCs also exhibit enhanced immunoreactivity for the iron exporter ferroportin and the iron-regulating storage mediator hepcidin (**F**). **G** The transmembrane iron channel DMT-1 is enriched at lesion borders, albeit with low intensity. **H** The transferrin receptor (TfR) is only detected on few glial cells, presumably astrocytes based on morphology, at the lesion rim (inset), while (**I**) its membranous expression on macrophages can be detected in another late active MS lesion core. **J** Similarly, the ferritin receptor Scara5 can hardly be detected at the iron-positive lesion rim, while (**K**) it is weakly expressed on macrophages in late active lesion cores. Scale bars: 50 µm (insets, panels I and K), 125 µm (all others)

**Fig. 2:**
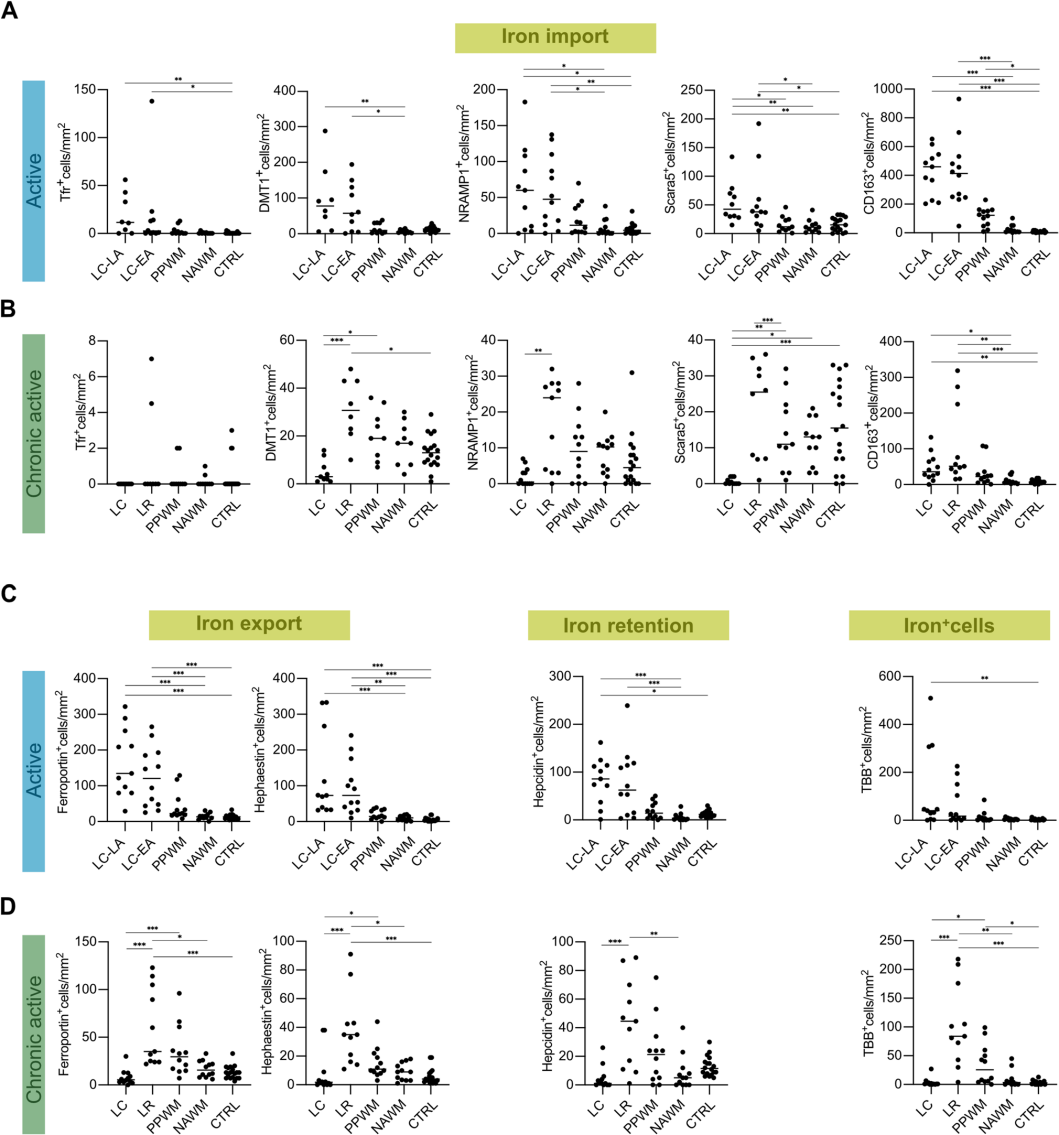
Data from manual counting of MCs, based on cellular morphology, featuring various proteins involved in iron import of active (**A**), chronic active (**B**) MS lesions and proteins involved in export and storage as well as iron itself, in active (**C**) and chronic active (**D**) lesions, together with corresponding MS NAWM and CTRL ROIs. Data are derived from FFPE cohort A, including 18 control samples and 24 MS cases with 12 active lesions, 11 chronic active lesions and one inactive lesion (see Supplementary Table 1). *CTRL* white matter of controls, *EA* early active, *LA* late active, *LC* lesion center, *LR* lesion rim, *NAWM* normal-appearing white matter, *PPWM* peri-plaque white matter, *ROI* region of interest. One star (*) corresponds to a *p* value of < 0.05, two stars (**) to < 0.01 and three stars to < 0.001

**Fig. 3:**
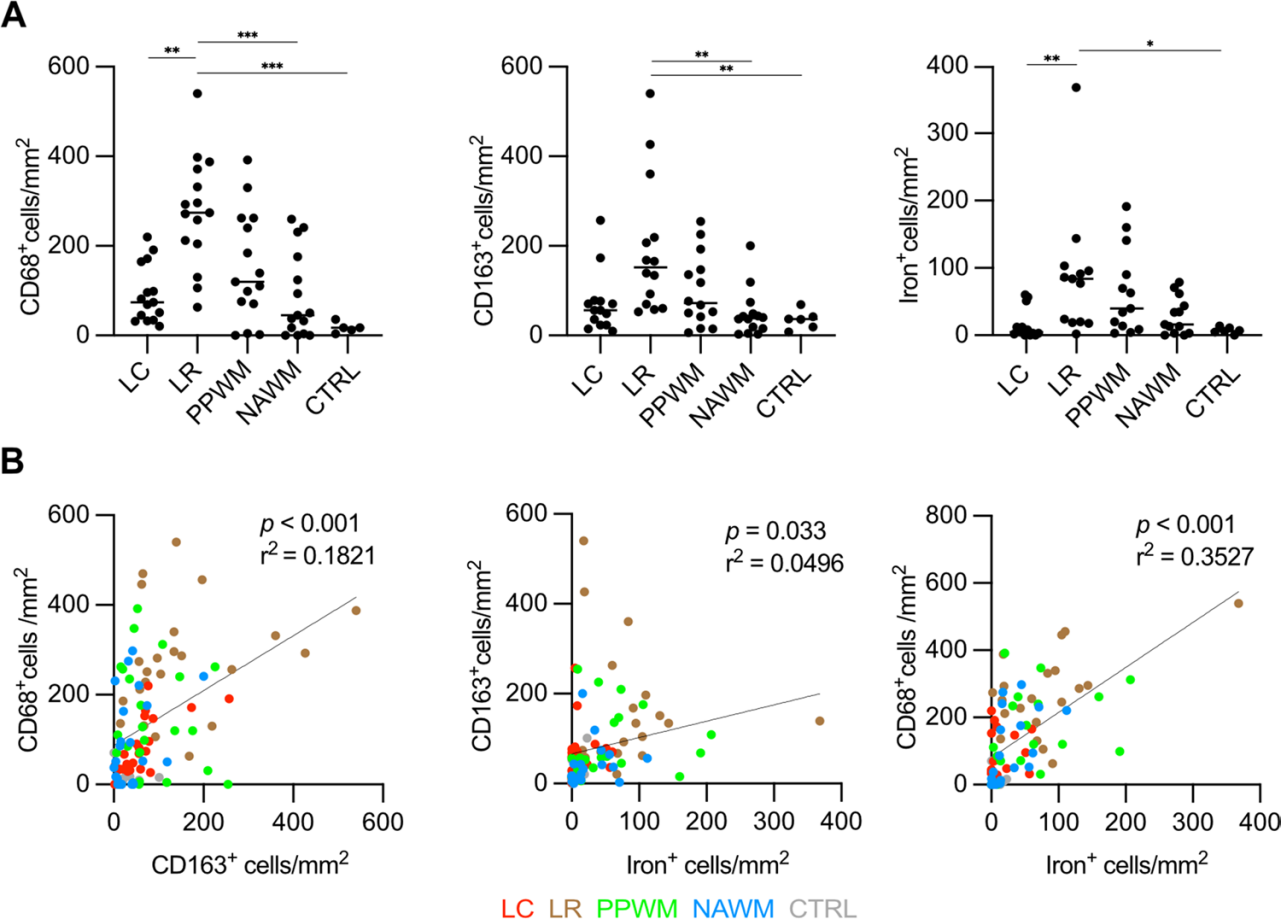
**A** Dot plots and **B** scatter plots of MC counts immunoreactive for CD68 and CD163 on protein level, as well as MCs accumulating iron itself, in chronic active lesions, NAWM and CTRL ROIs. The colors of data points in (**B**) indicate the different ROIs which are defined below the plots. Data are derived from frozen tissue samples of cohort B, including 6 control samples and 14 MS cases with 23 chronic active lesions (see Supplementary Table 1). *CTRL* white matter of controls, LC = lesion center, LR = lesion rim, NAWM = normal-appearing white matter of MS cases, *PPWM* peri-plaque white matter. One star (*) corresponds to a *p* value of < 0.05, two stars (**) to < 0.01 and three stars to < 0.001

In FFPE samples of the exploratory cohort A, we found accumulation of iron-laden MCs in late active lesion zones (LC-LA) and chronic active lesion rims (LR) (Figs. 1 and 2), as reported [24]. In LC-LA regions, this was paralleled by elevated MC numbers IHC-positive for the iron uptake markers TfR (TfR, *TFRC*), DMT1 (*SLC11A2*), NRAMP1 (*SLC11A1*), Scara5 (*SCARA5*) and – by far the highest—CD163, the scavenger receptor for hemoglobin-haptoglobin complexes. In LR regions of chronic active lesions, however, only DMT1 and CD163 were increased, when compared to either CTRL (DMT1 and CD163) or NAWM (only CD163) (Fig. 2). Of note, the transferrin receptor, while readily detectable on membranes of MCs in early active lesion zones (LC-EA) and LC-LA regions (Fig. 1I), was not enriched on MCs at LR regions. In addition, increased numbers of MCs immunoreactive for the iron exporter proteins ferroportin (*SLC40A1*) and hephaestin (*HEPH*) as well as the functional ferroportin antagonist hepcidin (*HAMP*) were found in LC-LA and LR regions, suggesting iron-related increase of ferroportin translation and its functional downregulation via hepcidin expression. Correlation analysis between MC counts of the mentioned iron importers and iron across samples with active and chronic active lesion ROIs revealed by far the strongest correlations between iron and CD163 (r^2^ = 0.4017, *p* < 0.001) as well as DMT1 (r^2^ = 0.4491, *p* < 0.001) in chronic active lesions (Supplementary Fig. 1). Due to the explorative approach in cohort A, not all stainings were available for the entire cohort. In some cases, active lesions only had early active and no late active zones, hence, resulting in different numbers of data points. In the confirmatory cohort B of frozen samples containing chronic active lesions only, we found increased numbers of CD68^+^ MCs at the LR vs. LC (*p* = 0.016), NAWM (*p* = 0.002) and CTRL (*p* = 0.001) (Fig. 3). CD163^+^ cells were more numerous at the LR, compared to NAWM (*p* = 0.001) and CTRL (*p* = 0.008). Iron-positive MCs were likewise more numerous at LR areas when compared to LC (*p* = 0.004) and CTRL (*p* = 0.028). Quantification levels correlated for CD163 and CD68 (r^2^ = 0.1821, *p* < 0.001), CD163 and iron (r^2^ = 0.0496, *p* = 0.033) and CD68 and iron (r^2^ = 0.3527, *p* < 0.001). These data suggest that MC iron uptake at MS lesion rims is less likely mediated by transferrin but rather driven through CD163 and haptoglobin-hemoglobin complexes.

### Upregulation of the CD163-HMOX1-HAMP pathway in MCs at MS lesion rims

**Fig. 4:**
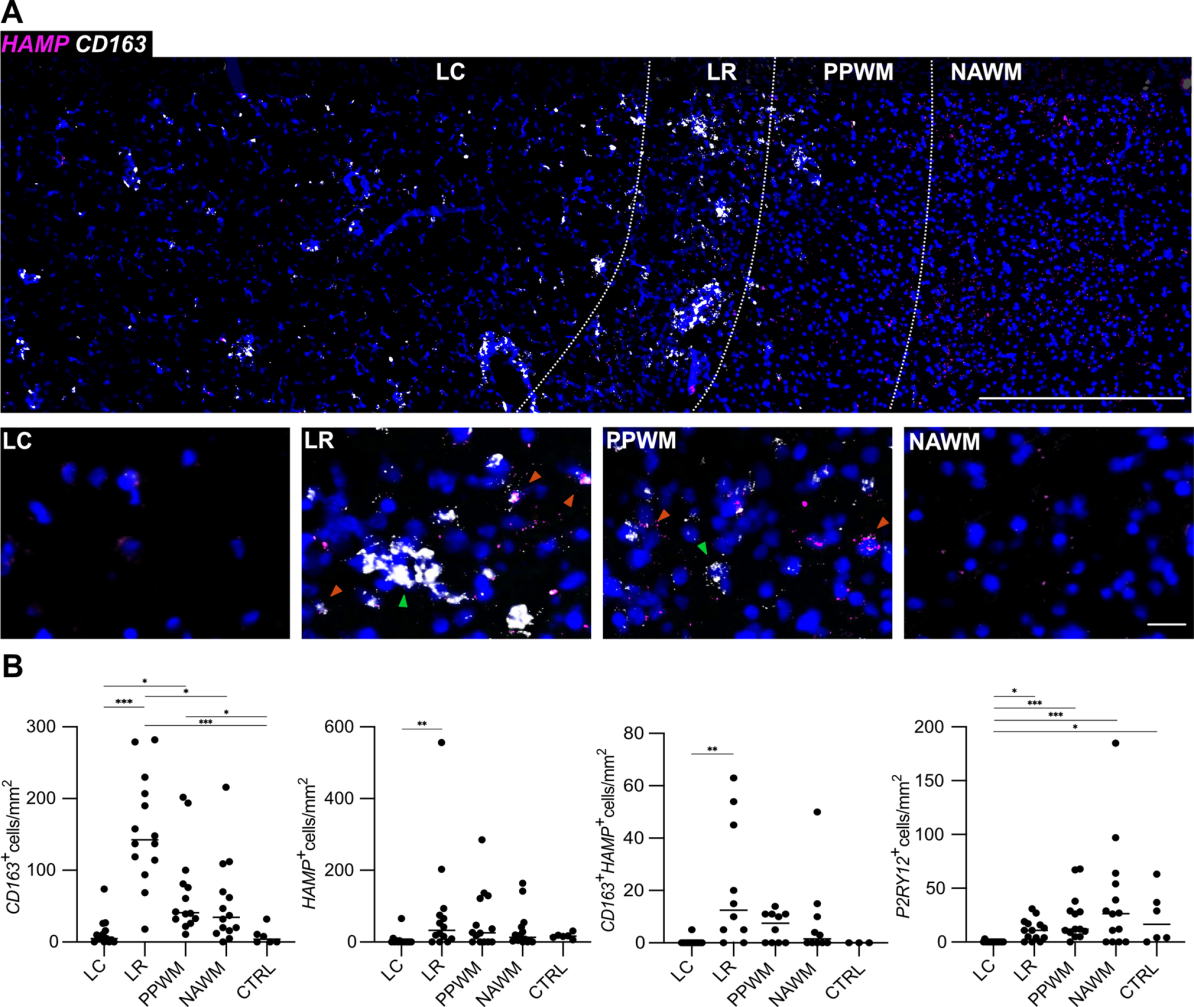
**A** Spatial expression of *HAMP* (magenta) and *CD163* (white) transcripts across a typical chronic active MS lesion based on multiplex smFISH; representative image (top) showing all 4 lesion areas (ROIs) including LC, LR, PPWM and NAWM with zoom-in images below (bottom row); note in zoom-in panels, colored arrows exemplify *HAMP*^+^*CD163*^+^ cells, and green arrows indicate *CD163*^+^ cells. Scale bars: 500 µm (top) and 25 µm (bottom row). **B** Dot plots depict the data derived from manual counting of smFISH signals. *LC* lesion center, *LR* lesion rim, *NAWM* normal-appearing white matter of MS cases, *PPWM* peri-plaque white matter. One star (*) corresponds to a *p* value of < 0.05, two stars (**) to < 0.01 and three stars to < 0.001

**Fig. 5:**
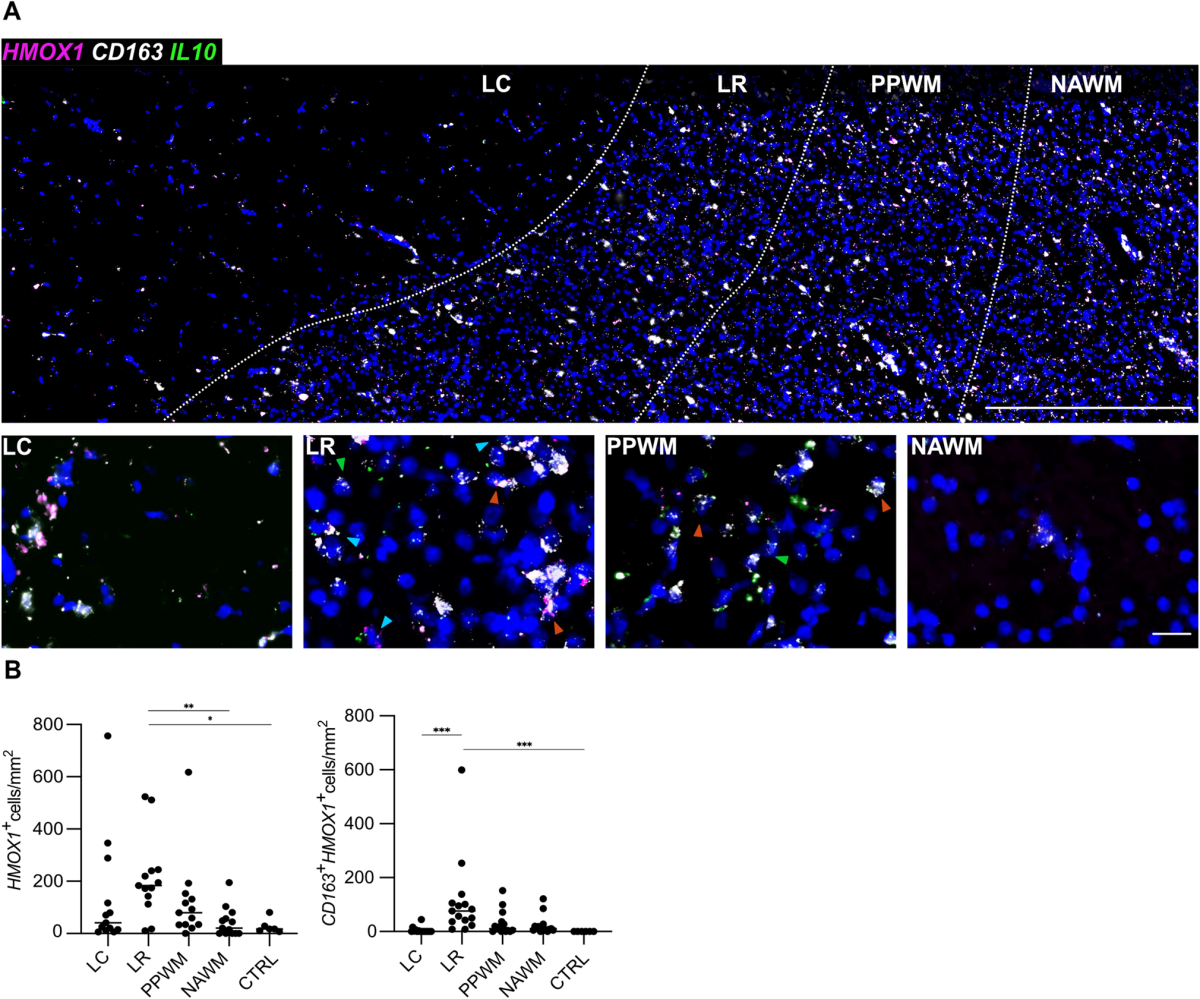
**A** Spatial expression of *HMOX1* (magenta), *CD163* (white) and *IL10* (green) transcripts across a typical chronic active MS lesion based on multiplex smFISH; representative image (top) showing all 4 lesion areas (ROIs) including LC, LR, PPWM and NAWM with zoom-in images below (bottom row); note in zoom-in panels light blue arrows indicate *HMOX1*^+^*CD163*^+^*IL10*^+^ cells, red arrows indicate *HMOX1*^+^*CD163*^+^ cells, and green arrows indicate *CD163*^+^ cells. **B** Dot plots depict the data derived from manual counting of smFISH signals. LC = lesion center, LR = lesion rim, NAWM = normal-appearing white matter of MS cases, PPWM = peri-plaque white matter. One star (*) corresponds to a *p* value of < 0.05, two stars (**) to < 0.01 and three stars to < 0.001

By RNA ISH on cohort B consisting of frozen samples, upregulation of *CD163* was detected at the LR compared to LC (*p* < 0.001), NAWM (*p* = 0.037) and CTRL (*p* = 0.001) areas (Fig. 4). Moreover, we detected increased numbers of *CD163*-expressing cells at PPWM areas compared to LC (*p* = 0.012) and CTRL (*p* = 0.050). Both *HAMP*-only (encoding hepcidin) (*p* = 0.010) and *CD163*/*HAMP-* (*p* = 0.007) co-expressing MCs were found to be increased at LR areas compared to LC. Conversely, *P2RY12*-expressing MCs, indicating a homeostatic MC phenotype [65], were found to be decreased at LC areas vs. CTRL (*p* = 0.005), NAWM (*p* < 0.001), PPWM (*p* < 0.001) and LR (*p* = 0.001), confirming previous data [65]. *HMOX1*, encoding heme oxygenase 1 that catalyzes heme to iron, carbon monoxide and biliverdin [11], was upregulated at the LR, compared to NAWM (*p* = 0.005) and CTRL (*p* = 0.033) (Fig. 5). Cells co-expressing *CD163* and *HMOX1* accumulated at the LR, compared to LC (*p* < 0.001) and CTRL (*p* < 0.001). Of note, numbers of *CD163*-expressing MCs correlated with *HMOX1* (r^2^ = 0.1495, *p* = 0.003) and *HAMP* expression (r^2^ = 0.0632, *p* = 0.010) as well as presence of iron-laden MCs (r^2^ = 0.2155, *p* < 0.001). Furthermore, *HAMP* expression and presence of iron-laden MCs correlated with each other (r^2^ = 0.1814, *p* < 0.001). Taken together, we could demonstrate that the *CD163-HMOX1-HAMP* axis was upregulated in MCs at chronic active lesion rims in a spatially restricted pattern.

### Expression of IL-10 and C1QA in CD163^+^ MCs at chronic lesion rims

**Fig. 6:**
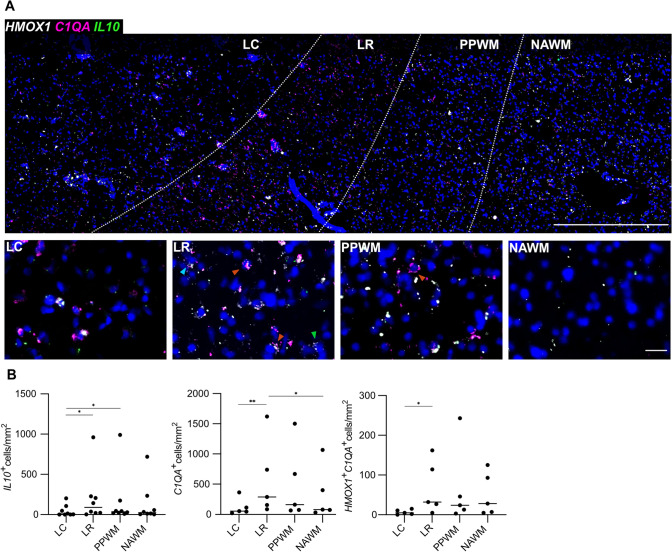
**A** Spatial expression of *HMOX1* (white), *C1QA* (pink) and *IL10* (green) transcripts across a typical chronic active MS lesion based on multiplex smFISH; representative image (top) showing all 4 lesion areas (ROIs) including LC, LR, PPWM and NAWM with zoom-in images below (bottom row); note light blue arrow indicates an *IL10*^+^*C1QA*^+^*HMOX*^+^ cell, red arrows indicate *C1QA*^+^*HMOX*^+^ double-positive cells and the green arrow an *HMOX1*^+^ cell. **B** Dot plots depict the data derived from manual counting of smFISH signals. *LC* lesion center, *LR* lesion rim, *NAWM* normal-appearing white matter of MS cases, *PPWM* peri-plaque white matter. One star (*) corresponds to a *p* value of < 0.05 and two stars (**) to < 0.01

To gain further insight into the functional properties of *CD163*-expressing MCs at MS lesion rims, we focused on *IL10* and *C1QA* expression as two signature markers for regulatory (*IL10*) [41] and pro-inflammatory (*C1QA*) functions [1, 16, 33, 52]. *C1QA* expression was elevated at the LR vs. LC (*p* = 0.0087) and NAWM (*p* = 0.042), as described [1] (Fig. 6). Next, we assessed co-expression with *CD163* and found that *C1QA* co-expressing cells were increased at the LR vs. LC (*p* = 0.042) (Fig. 6), while* IL10* co-expressing cells were upregulated at PPWM vs. LC (*p* = 0.030). These findings suggest that both pro-inflammatory and, to a certain extent, regulatory functions are associated with *CD163* expression at spatially restricted lesion border areas in MS (Supplementary Fig. 2).

### Paramagnetic rim lesions are linked to clinical disability

**Fig. 7:**
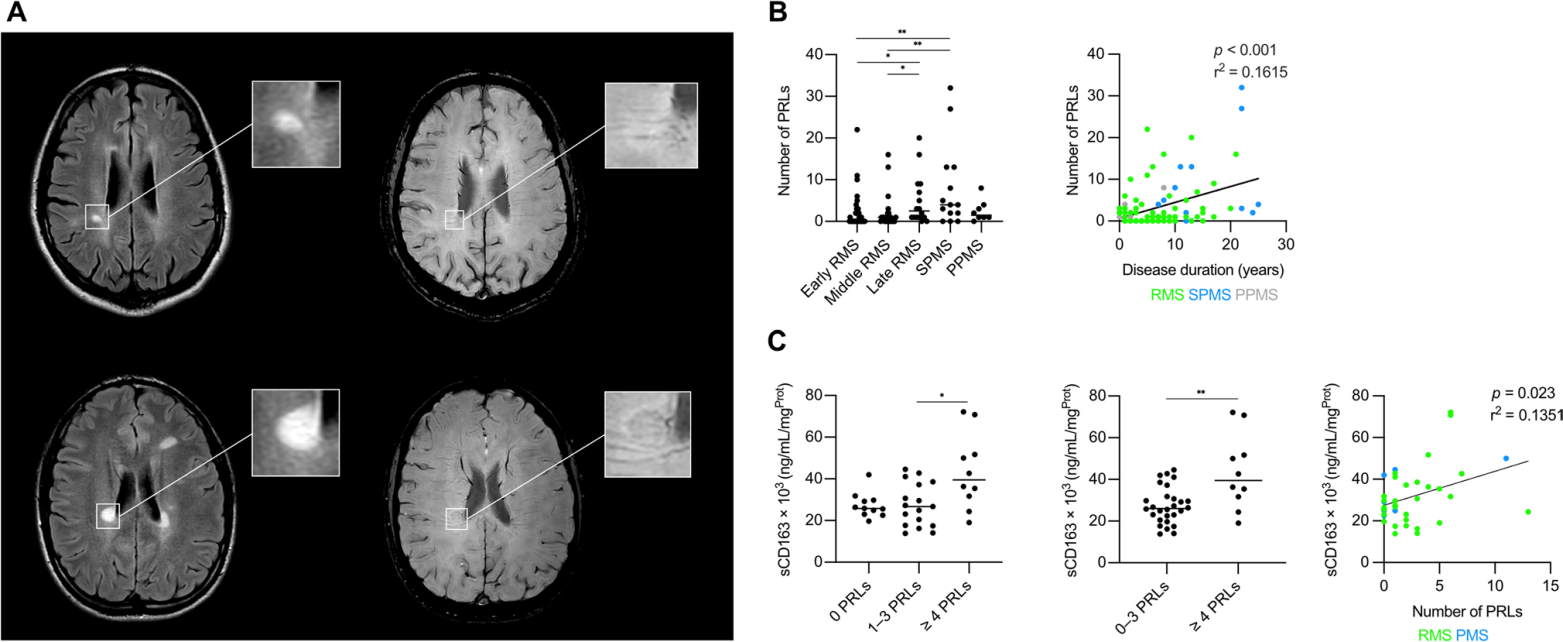
**A** MR images showing that PRLs (bottom images) versus non-PRLs (top images) are characterized by a hypointense paramagnetic rim on susceptibility-weighted imaging (SWI, right images) surrounding the FLAIR-hyperintense lesion core (left images). **B** The number of PRLs increases in the late RMS and SPMS phase (left panel) and is correlated with disease duration (right panel). **C** pwMS with ≥ 4 PRLs have significantly higher sCD163 levels in the CSF correlating with the number of PRLs on MRI. *CSF* cerebrospinal fluid, *PMS* progressive MS, *PPMS* primary-progressive MS, *PRL* paramagnetic rim lesion, *RMS* relapsing MS, sCD163 = soluble cluster of differentiation 163, *SPMS* secondary-progressive MS. One star (*) corresponds to a *p* value of < 0.05 and two stars (**) to < 0.01

A total of 341 PRLs in 61 pwMS (62.2%) were analyzed; 37 pwMS (37.8%) had no PRLs. Median number of PRLs per individual was 1 (IQR 0–4). PRLs were more commonly seen in men (75.6% vs. 52.6%, *p* = 0.034) with longer disease duration (8 [2.5–13] vs. 4 [2.5–7.5], *p* = 0.034) and higher disability scores measured by EDSS (2.5 [1.4–4.0] vs. 1.0 [0–2.8], *p* = 0.002) and MSSS (3.65 [1.74–5.87] vs. 2.34 [0.45–5.35], *p* = 0.020). Male sex (OR 3.83; 95% CI 1.44, 10.19; *p* = 0.007) and longer disease duration (OR 1.10; 95% CI 1.00, 1.22; *p* = 0.044) were both independently associated with higher risk for the PRL presence, whereas age, EDSS and Hp genotype were not. Individuals with early (0 [0–2]) and intermediate RMS phases (1 [0–2]) had significantly lower number of PRLs compared to pwMS in the late RMS phase (2.5 [1–8.5]) (*p* = 0.013 and *p* = 0.027, respectively) (Fig. 7). No differences in PRL counts were observed between pwMS with RMS and SPMS (4 [1.5–13], *p* = 0.606). Among pwMS without PRLs, only 4 (10.8%) had PMS (*p* = 0.045) (Table 2). The number of PRLs correlated with the age of pwMS (*r*_s_ = 0.2264, *p* = 0.025), disease duration (*r*_s_ = 0.2774, *p* = 0.006), EDSS (r_s_ = 0.4036, *p* < 0.001), and MSSS (r_s_ = 0.2620, *p* = 0.010). In a multivariate linear regression model, the number of PRLs was associated with EDSS (β = 0.22; 95% CI 0.04, 1.34; *p* = 0.039) and disease duration (β = 0.31; 95% CI 0.10, 0.51; *p* = 0.003) but not with age, sex or *Hp* haplotype of pwMSs (Fig. 7). Sensitivity analyses removing individuals from the Mannheim MRI cohort did not significantly change the overall results or impact of single variables (data not shown).

**Table 2:** Clinical data of the study cohort according to their haptoglobin genotype

	Hp1-1 (n = 8)	Hp2-2 and Hp2-1 (n = 90)	p-value
Female^a^	5 (62.5)	52 (57.8)	> 0.999
Age (years)^b^	31.9 (3.9)	38.5 (10.8)	0.088
Disease duration (years)^c^	7 (3–11.8)	6 (2–10.5)	0.800
EDSS^c^	0.5 (0–1.8)	2.0 (1.0–3.5)	**0.041**
MSSS^c^	0.56 (0.34–3.84)	3.17 (1.28–5.87)	**0.038**
Presence of PRLs^a^	4 (50.0)	57 (63.3)	0.471
Number of PRLs^c^	1.5 (0–4.5)	1 (0–4)	0.883
RMS (pwMS)^a^	7 (87.5)	69 (76.7)	0.679

Numbers in boldface highlight significant p-values

^a^Number (percentage), ^b^Mean and standard deviation, ^c^Median and interquartile range

*EDSS* expanded disability status scale, *MSSS* multiple sclerosis severity scale, *PRL* paramagnetic rim lesion, *RMS* relapsing multiple sclerosis

### *Hp2-1* and *Hp2-2* haplotypes are associated with clinical disability

To gain insight into the relationship between Hp haplotype and clinical as well as MRI parameters, we analyzed DNA samples from pwMS: *Hp1-1* (n = 8, 8.2%), *Hp2-2* (n = 39, 39.8%) and *Hp2-1* (n = 51, 52.0%). Of note, this distribution was in Hardy–Weinberg equilibrium [45]. pwMS with *Hp2-2* and *Hp2-1* pooled genotypes showed higher EDSS (2.0 [1.0–3.5] vs. 0.5 [0–1.8]; *p* = 0.041) and MSSS levels (3.17 [1.27–3.87] vs. 0.56 [0.34–3.84]; *p* = 0.038) compared to pwMS with an *Hp1-1* haplotype. Differences between the Hp haplotype in age (38.5 [10.8] vs. 31.9 [3.9], *p* = 0.088), sex (52 [57.8] *vs.* 5 [62.5], *p* > 0.999), disease duration (6 [2–10.5] vs. 7 [3–11.8], *p* = 0.800), RMS phase (69 [76.7] *vs.* 7 [87.5], *p* = 0.679), and the number of PRLs were not significant (1 [0–4] *vs.* 1.5 [0–4.5], *p* = 0.883).

### CSF sCD163

**Fig. 8:**
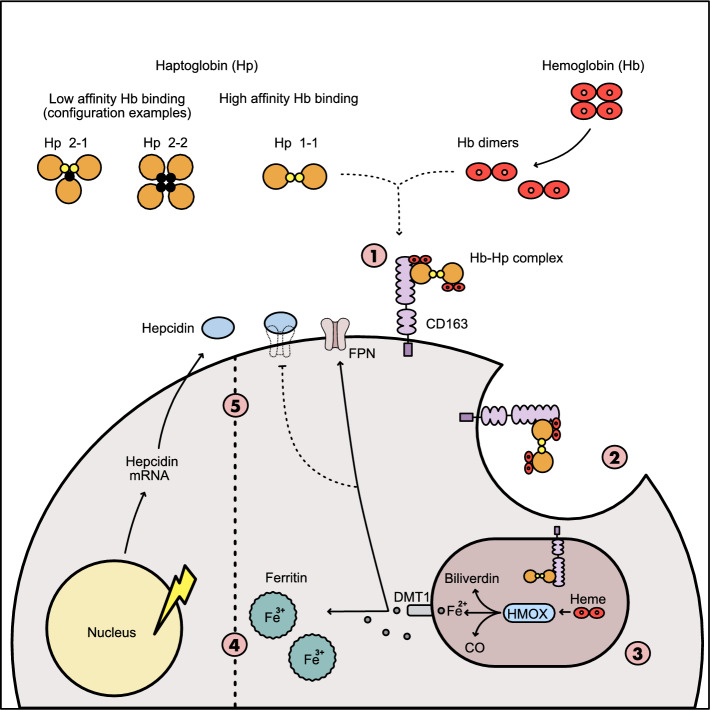
Schematic summarizes putative mode of action of iron uptake and metabolism mechanisms in iron-laden MCs at chronic active MS lesion rims: (**1**) Binding of haptoglobin-hemoglobin to CD163, a high-affinity scavenger receptor, at the surface of a lesion rim-associated MC; (**2**) internalization of haptoglobin-hemoglobin bound CD163 complex, and (**3**) enzymatic breakdown of heme to form biliverdin through heme oxygenase (HMOX, encoded by *HMOX1*) function; (**4**) transport of ferrous iron (Fe^2+^) through DMT1 (encoded by *SLC11A2*) function that is either oxidized to Fe^3+^ and bound to ferritin or (**5**) directly exported into extracellular spaces through ferroportin (FPN, encoded *SLC40A1*) function; note that ferroportin function is regulated by hepcidin (encoded by *HAMP*) that binds to ferroportin and inhibits iron export through internalization

In 38 pwMS with available CSF sample, sCD163 measurements were performed. The median CSF sCD163 concentration was 28.3 ng/mL (22.9–39.2). pwMS with $\ge$ 4 PRLs had higher CSF sCD163 levels than pwMS with 1–3 PRLs (39.5 [29.9–56.5] *vs.* 26.7 [17.6–37.9], *p* = 0.047) (Fig. 7). As no differences in the sCD163 concentration between pwMS without PRLs (25.8 [23.0–29.7]) and pwMS with 1–3 PRLs were seen (*p* > 0.999), those were pooled. pwMS with $\ge$ 4 PRLs had significantly higher sCD163 levels than individuals with $\le$ 3 PRLs (*p* = 0.009). CSF sCD163 concentration correlated with the number of PRLs (r^2^ = 0.1351, *p* = 0.023) but not with age, disease duration or EDSS (Fig. 7). After excluding two outliers from the analysis, statistical significance was lost, yet a trend towards higher sCD163 in pwMS with $\ge$ 4 PRLs was still present (*p* = 0.059) Fig. 8.

## Discussion

In this combined histopathological *postmortem* and *in-vivo* double center, cross-sectional study, we aimed to decipher molecular and cell type-specific pathways underlying MC iron uptake and sustained iron storage at the rim of chronic active MS lesions. Further, we aimed to provide a link between a putative genetic risk factor for iron rim formation, which is the association between *Hp* haplotypes and the presence of PRLs in pwMS. Iron accumulation in MCs at the edge of chronic active lesions is now widely accepted as the pathological substrate of PRLs seen in the MRI of pwMS [50]. This iron has been suggested to arise from degenerating, iron-containing oligodendrocytes during demyelination at the edge of these lesions [4, 24]. However, oligodendrocytes in the periplaque WM around chronic active lesions were found to contain reduced amounts of iron [49], which might be related to the upregulation of iron-exporting ferroxidases on oligodendrocytes around WM lesions [20]. We therefore hypothesize that, in addition, other iron import mechanisms might be responsible for iron accumulation in PRLs in pwMS, with a particular emphasis on active scavenging via the hemoglobin-haptoglobin receptor CD163 and its downstream pathway in MC subtypes.

To gain insight into the cell type-specific expression of various iron uptake, storage and export markers, we performed a histopathological study using two independent exploratory FFPE and confirmatory frozen tissue cohorts from *postmortem* MS cases analyzed by protein IHC and RNA ISH. We could demonstrate that the transferrin receptor is hardly present on MCs at iron rims, indicating that transferrin is not among the main sources for iron uptake in rim-associated MCs. Furthermore, neither the ferritin receptor Scara5 nor the transmembrane importer channel NRAMP1 were upregulated on rim-related MCs in MS lesions. Conversely, both DMT1 and the hemoglobin-haptoglobin complex receptor CD163 were significantly upregulated on a subtype of MCs at MS lesion rims and also strongly correlated with iron-positive MCs across ROIs of cases containing chronic active lesions. These findings suggest that at least part of the lesion rim-associated iron might derive from hemoglobin-bound iron. These data are in line with a study on immortalized microglial cells, showing that pro-inflammatory activation using LPS promoted non-transferrin-bound microglial iron uptake pathways including up-regulation of DMT1, while anti-inflammatory polarization using IL4 activation promoted iron uptake via the transferrin receptor pathway [37]. In the non-lesioned healthy brain, CD163 is only expressed at detectable levels on perivascular macrophages [8, 64]. Expression of CD163 on resident microglia and infiltrating macrophages has been described under various pathological conditions in the CNS parenchyma, including HIV encephalitis [8], subarachnoid hemorrhage [22], and in active [46, 65] as well as rims of chronic active MS lesions [64, 65]. This expression partially correlated with MC iron accumulation, pro-inflammatory activation status as signified by HLA-DR expression, and expression of ADAM17 in case of subarachnoid hemorrhage [22]. CD163 upregulation could also be induced by experimental incubation with haptoglobin-hemoglobin complexes [8]. Thus, CD163 upregulation in MCs would, at least partially, indicate a compromised blood–brain barrier, leading to influx of haptoglobin-hemoglobin complexes among other factors such as fibrinogen [36, 47], from the blood into the CNS parenchyma and leading to microglial activation, as shown by HLA-DR upregulation and cellular morphological alterations [8].

Of note, we also observed that the soluble form of CD163, sCD163 [36], was enriched in the CSF of pwMS and correlated with the number of PRLs, suggesting that sCD163 might represent a potential biomarker reflecting PRL status and clinical disability in MS. sCD163 is shed from membrane-bound CD163 by ADAM17 [18, 22], a metallopeptidase. We therefore suggest that the correlation of CSF sCD163 levels with PRL numbers supports our interpretation that iron accumulation at rims of chronic active lesions involves CD163-mediated iron uptake. Hemoglobin contains by far the largest pool of iron in the human body [42]. A previous study reported increased levels of liberated, cell-free hemoglobin in pwMS, when compared with controls [31]. In addition, the blood–brain barrier is subtly and chronically compromised at edges of chronic active lesions [25], opening the possibility of slow cell-free hemoglobin-haptoglobin complex diffusion into the CNS parenchyma over several years of PRL persistence. Is therefore hemoglobin-haptoglobin uptake via CD163 a critical determinant for MC-related iron persistence at lesion rims in pwMS?

To address this question, we investigated whether the haptoglobin genotype would be associated with clinical disability and PRLs in pwMS. Although we could not provide evidence for a genetic risk in association with the presence of PRLs, probably also limited by the small sample size, we found that the *Hp2-1* and *Hp2-2* haplotypes were associated with slightly aggravated clinical disability. It is known that different Hp haplotypes are linked to different protein sizes that could influence binding and uptake of these complexes by CD163. Similar to the relationship with the *Hp* genotype, we found that the number of PRLs was associated with clinical disability, specifically, higher EDSS and MSSS scores but not with age and disease duration. Hence, our findings suggest that the presence of PRLs and *Hp2-1/2–2* haplotypes might be critical determinants predicting clinical disability in pwMS. However, our findings are exploratory in nature and could have been affected by slightly yet not significantly older pwMS with the *Hp-2–1* and *Hp2-2* haplotypes, which is why they should be interpreted cautiously.

Next, we aimed at deciphering the iron-metabolizing mechanisms downstream of CD163-mediated iron uptake. Of note, we found that *HMOX1* was strongly upregulated in *CD163* co-expressing MCs at chronic active lesion edges. HMOX1 is the inducible and rate-limiting enzyme in heme catabolism, also expressed by glial cells and capable of degrading heme to iron, biliverdin and carbon monoxide [51]. Heme is found as a prosthetic group in a variety of enzymes and hemoglobin, where it is responsible for oxygen binding [17]. HMOX1 has been shown to be expressed by MCs in active and chronic active lesions in MS [32, 57]. In our view, concomitant up-regulation of *HMOX1* in MCs expressing *CD163* supports the interpretation that this receptor is actively scavenging hemoglobin, which we therefore propose to be among the major sources of iron in MCs at iron-accumulating MS lesion edges. Further, we observed that *HAMP* encoding hepcidin, a key regulator of iron export into the circulation, was upregulated in MCs at MS lesion areas suggesting that imported iron is actively kept in MCs preventing release into the surrounding tissue environment.

To gain more insight into the downstream functional properties of iron-metabolizing MCs at MS lesion rims, we investigated the expression of *P2RY12*, as a classic homeostatic MC marker in brain tissues, as compared to *C1QA*, as a classic pro-inflammatory marker indicating complement production in MCs [1]. We found reduced spatial expression levels for *P2RY12* at MS lesion rim areas, confirming previous data [65] and indicating a shift towards a pro-inflammatory MC phenotype [40, 65, 66]. In addition, we noted that the tissue-regulatory cytokine *IL10* appeared to be upregulated in a subtype of MCs in PPWM areas, hence, in more distance to the pro-inflammatory LC and LR, where *C1QA* and *CD163* appeared to show the strongest upregulation. Of note, IL10 is a potent macrophage deactivation factor suppressing the release of macrophage-derived TNF, reactive oxygen species and others [7]. These findings suggest a complex, however, spatially well-orchestrated interplay between MC subtypes with signatures related to iron uptake and immune cell activation enhanced at LC and LR areas, whereas events related to iron persistence and tissue regulation were more prominent in PPWM areas in further distance to the inflamed regions of interest.

The proposed PRL formation via uptake of haptoglobin-hemoglobin-bound complexes into MCs does not seem to be strongly influenced by alterations of the peripheral iron status though, since the levels of serum hemoglobin, free serum hemoglobin and transferrin-bound serum iron were not significantly elevated in pwMS having ≥ 1 PRLs, when compared to pwMS with 0 PRLs or controls [28]. Moreover, serum hepcidin levels were not significantly elevated in pwMS compared to controls [9]. Taken together, these results suggest that PRL formation might largely depend on local blood–brain barrier integrity and chronic lesion activity rather than variations in blood iron levels or total hemoglobin status, although larger cohorts are needed to settle these assumptions.

To conclude, the current study provides new insight into the pathophysiological mechanisms behind PRL formation and MC-related iron uptake in pwMS, which might depend on the binding of haptoglobin-hemoglobin complex to CD163. Future work needs to demonstrate whether a combination of CSF markers, MR imaging and genetic risk parameters can be used as biomarkers to stratify pwMS into subgroups with higher risk for iron rim lesion formation and clinical detoriation. Furthermore, as new therapies such as Bruton tyrosine kinase (BTK) inhibitors specifically target MCs including brain-resident microglia and blood-derived monocytes, a better understanding of MC biology will be critical for the development of new interventional therapies in MS [30]. Our and future findings will therefore help develop personalized treatment strategies to specifically target MCs at lesion rims, at least in a subgroup of pwMS.

### Supplementary Information

Below is the link to the electronic supplementary material.

Supplementary file1 (DOCX 24 KB)

Supplementary file2 (TIFF 953 KB)

Supplementary file3 (TIFF 473 KB)

## Data Availability

Most data generated or analyzed have been included in the main manuscript and supplementary material. Additional data including raw histopathological and MR imaging data are available from the corresponding authors upon request.
