# Supplementary material for: Myeloid cell iron uptake pathways and paramagnetic rim formation in multiple sclerosis
Source: Acta Neuropathol. 2023 Sep 16;146(5):707–24. doi: 10.1007/s00401-023-02627-4 (PMC10564819; doi:10.1007/s00401-023-02627-4)
Supplement: Supplementary file 1 — Supplementary file1 (DOCX 24 KB) [file 401_2023_2627_MOESM1_ESM.docx]

**Supplementary Figure 1:** Exploratory correlation analyses of manual countings of MCs positive for iron importers (y-axis) and iron (x-axis), stratified for (**A**) active and (**B**) chronic active MS lesions. Correction for multiple testings has not been performed. The same dataset as shown in **Figure 2** (FFPE cohort A). CTRL = white matter of controls, EA = early active, LA = late active, LC = lesion center, LR = lesion rim, NAWM = normal-appearing white matter of MS cases, PPWM = peri-plaque white matter.

**Supplementary Figure 2:** Correlation analysis of manual countings based on smFISH stainings showing the relation between *C1QA*, *CD163* and *IL10* expression across MS lesion areas. Correction for multiple testings has not been performed.

**Supplementary Table 1.**

| **Case** | **Age (years), Median (range)** | **Sex, F:M ratio** | **Clinical course** | **Disease duration (months), Median (range)** | **Lesion type** | **Regions of interest (ROI)** |
| --- | --- | --- | --- | --- | --- | --- |
| Controls FFPE Sample A (1- 18) | 67.5 (30-97) | 12:6 | - | - | - | 18 CTRL |
| MS cases FFPE Sample A (MS 1 - 24) | 49 (34-78) | 12:12 | 9 AMS, 3 RRMS, 7 SPMS, 5 PPMS | 120 (0.2 - 492) | 12 A, 11 CA, 1 I | 11 LC-LA 12 LC-EA, 12 LC-I, 11 LR, 24 PPWM, 24 NAWM |
| MS 1 | 45 | M | AMS | 0.2 | 1 A | 1 LC-EA, 1 PPWM, 1 NAWM |
| MS 2 | 45 | M | AMS | 0.6 | 1 A | 1 LC-LA, 1 LC-EA, 1 PPWM, 1 NAWM |
| MS 3 | 35 | M | AMS | 1 .5 | 1 A | 1 LC-LA, 1 LC-EA, 1 PPWM, 1 NAWM |
| MS 4 | 52 | M | AMS | 1 . 5 | 1 A | 1 LC-LA, 1 LC-EA, 1 PPWM, 1 NAWM |
| MS 5 | 69 | F | AMS | 2 | 1 A | 1 LC-LA, 1 LC-EA, 1 PPWM, 1 NAWM |
| MS 6 | 78 | M | AMS | 2 | 1 A | 1 LC-LA, 1 LC-EA, 1 PPWM, 1 NAWM |
| MS 7 | 46 | F | AMS | 3 | 1 A | 1 LC-LA, 1 LC-EA, 1 PPWM, 1 NAWM |
| MS 8 | 34 | F | AMS | 4 | 1 A | 1 LC-LA, 1 LC-EA, 1 PPWM, 1 NAWM |
| MS 9 | 46 | F | AMS | 7 | 1 A | 1 LC-LA, 1 LC-EA, 1 PPWM, 1 NAWM |
| MS 10 | 40 | F | RRMS | 120 | 1 A | 1 LC-LA, 1 LC-EA, 1 PPWM, 1 NAWM |
| MS 11 | 57 | F | RRMS | 156 | 1 A | 1 LC-LA, 1 LC-EA, 1 PPWM, 1 NAWM |
| MS 12 | 44 | F | RRMS | 262 | 1 A | 1 LC-LA, 1 LC-EA, 1 PPWM, 1 NAWM |
| MS 13 | 34 | M | SPMS | 120 | 1 CA | 1 LC, 1 LR, 1 PPWM, 1 NAWM |
| MS 14 | 41 | M | SPMS | 137 | 1 CA | 1 LC, 1 LR, 1 PPWM, 1 NAWM |
| MS 15 | 53 | F | SPMS | 241 | 1 CA | 1 LC, 1 LR, 1 PPWM, 1 NAWM |
| MS 16 | 76 | M | SPMS | 372 | 1 CA | 1 LC, 1 LR, 1 PPWM, 1 NAWM |
| MS 17 | 61 | F | SPMS | 396 | 1 CA | 1 LC, 1 LR, 1 PPWM, 1 NAWM |
| MS 18 | 46 | F | SPMS | 444 | 1 I | 1 LC, 1 PPWM, 1 NAWM |
| MS 19 | 59 | F | SPMS | 492 | 1 CA | 1 LC, 1 LR, 1 PPWM, 1 NAWM |
| MS 20 | 36 | M | PPMS | 61 | 1 CA | 1 LC, 1 LR, 1 PPWM, 1 NAWM |
| MS 21 | 67 | M | PPMS | 87 | 1 CA | 1 LC, 1 LR, 1 PPWM, 1 NAWM |
| MS 22 | 62 | M | PPMS | 144 | 1 CA | 1 LC, 1 LR, 1 PPWM, 1 NAWM |
| MS 23 | 53 | M | PPMS | 168 | 1 CA | 1 LC, 1 LR, 1 PPWM, 1 NAWM |
| MS 24 | 77 | F | PPMS | 168 | 1 CA | 1 LC, 1 LR, 1 PPWM, 1 NAWM |
| Controls Sample B (19 – 24) | 72.5 (54 – 81) | - | - | - | - | - |
| MS cases frozen sample B (25-41) | 50.50 (40 – 65) | 8:6 | 13 SPMS, 1 PPMS | 300 (72 – 480) | 23 CA | 23 LC, 23 LR, 23 PPWM, 23 NAWM |
| MS 25 | 42 |  | PPMS | 72 | 1 CA | 1 LC, 1 LR, 1 PPWM, 1 NAWM |
| MS 26 | 52 | F | SPMS | NA | 1 CA | 1 LC, 1 LR, 1 PPWM, 1 NAWM |
| MS 27 | 53 | M | SPMS | 192 | 1 CA | 1 LC, 1 LR, 1 PPWM, 1 NAWM |
| MS 28 | 40 | M | SPMS | 192 | 1 CA | 1 LC, 1 LR, 1 PPWM, 1 NAWM |
| MS 29 | 50 | F | SPMS | 276 | 2 CA | 2 LC, 2 LR, 2 PPWM, 2 NAWM |
| MS 30 | 61 | M | SPMS | 348 | 2 CA | 2 LC, 2 LR, 2 PPWM, 2 NAWM |
| MS 31 | 48 | F | SPMS | 348 | 1 CA | 1 LC, 1 LR, 1 PPWM, 1 NAWM |
| MS 32 | 65 | F | SPMS | 432 | 1 CA | 1 LC, 1 LR, 1 PPWM, 1 NAWM |
| MS 33 | 60 | F | SPMS | 348 | 1 CA | 1 LC, 1 LR, 1 PPWM, 1 NAWM |
| MS 34 | 51 | M | SPMS | 216 | 3 CA | 3 LC, 3 LR, 3 PPWM, 3 NAWM |
| MS 35 | 45 | F | SPMS | 300 | 1 CA | 1 LC, 1 LR, 1 PPWM, 1 NAWM |
| MS 36 | 42 | M | SPMS | 252 | 1 CA | 1 LC, 1 LR, 1 PPWM, 1 NAWM |
| MS 37 | 65 | F | SPMS | 480 | 1 CA | 1 LC, 1 LR, 1 PPWM, 1 NAWM |
| MS 38 | 50 | M | SPMS | 348 | 1 CA | 1 LC, 1 LR, 1 PPWM, 1 NAWM |

Table legend: A = active lesion, I = Inactive lesion, AMS = acute multiple sclerosis, CA = chronic active lesion, F = female, LC = inactive lesion center, LC-EA = early active lesion center, LC-LA = late active lesion center, LR = lesion rim, M = male, MS = multiple sclerosis, NAWM = normal-appearing white matter, PPMS = primary progressive multiple sclerosis, PPWM = periplaque white matter, CTRL = control white matter, RRMS = relapsing-remitting multiple sclerosis, SPMS = secondary progressive multiple sclerosis.

**Supplementary Table 2.**

| **Target** | **Origin** | **Dilution** | **Pretreatment** | **Source (product number)** | **Protocol** |
| --- | --- | --- | --- | --- | --- |
| PLP | Mouse monoclonal | 1:1,000 | EDTA, pH 9.0  60 min | AbD Serotec  (MCA839G) | FFPE IHC |
| CD163 | Mouse  monoclonal | 1:1,000 | Citrate pH 6.0  60 min | Novocastra  (NCL-L-CD163) | FFPE IHC |
| DMT1 | Rabbit polyclonal | 1:10,000 CSA | EDTA pH 9.0  60 min | Alpha Diagnostic (NRAMP23-S) | FFPE IHC |
| Ferroportin | Rabbit polyclonal | 1:200  CSA | EDTA pH 9.0 60 min | Abcam (ab78066) | FFPE IHC |
| Hepcidin | Rabbit polyclonal | 1:6,000  CSA | Citrate pH 6.0 60 min | Abcam (ab81010) | FFPE IHC |
| Hephaestin | Goat polyclonal | 1:50 | Citrate pH 6.0 60 min | Santa Cruz  (sc-49969) | FFPE IHC |
| NRAMP1 | Rabbit polyclonal | 1:1,000 | EDTA pH 9.0 60 min | LSBio  (LS-B9344) | FFPE IHC |
| Scara5 | Rabbit polyclonal | 1:200 | EDTA pH 9.0 60 min | Abcam (ab118894) | FFPE IHC |
| TfR | Mouse monoclonal | 1:500 | EDTA pH 9.0 60 min | Invitrogen (13-6800) | FFPE IHC |
| CD68 | Mouse  monoclonal | 1:100 | EDTA pH 9.0  60 min | Dako  (M0814) | FFPE IHC |
| CD68 | Mouse  monoclonal | 1:200 | PBST | Millipore (514H12) | FF IHC |
| MOG | Mouse  monoclonal | 1:1.000 | PBST | Bio-Rad (8-18C5) | FF IHC |
| CD163 | Mouse  monoclonal | 1:1,000 | Acetone 4°C 10 min | Novocastra  (NCL-L-CD163) | FF IHC |

Table legend: EDTA = [ethylenediaminetetraacetic acid,](https://en.wikipedia.org/wiki/Ethylenediaminetetraacetic_acid) FF = fresh frozen, FFPE = formalin-fixed paraffin-embedded, IHC = immunohistochemistry, PBST = phosphate-buffered saline with triton X
