# Supplementary figures and images for: Myeloid cell iron uptake pathways and paramagnetic rim formation in multiple sclerosis
Source: Acta Neuropathol. 2023 Sep 16;146(5):707–24. doi: 10.1007/s00401-023-02627-4 (PMC10564819; doi:10.1007/s00401-023-02627-4)

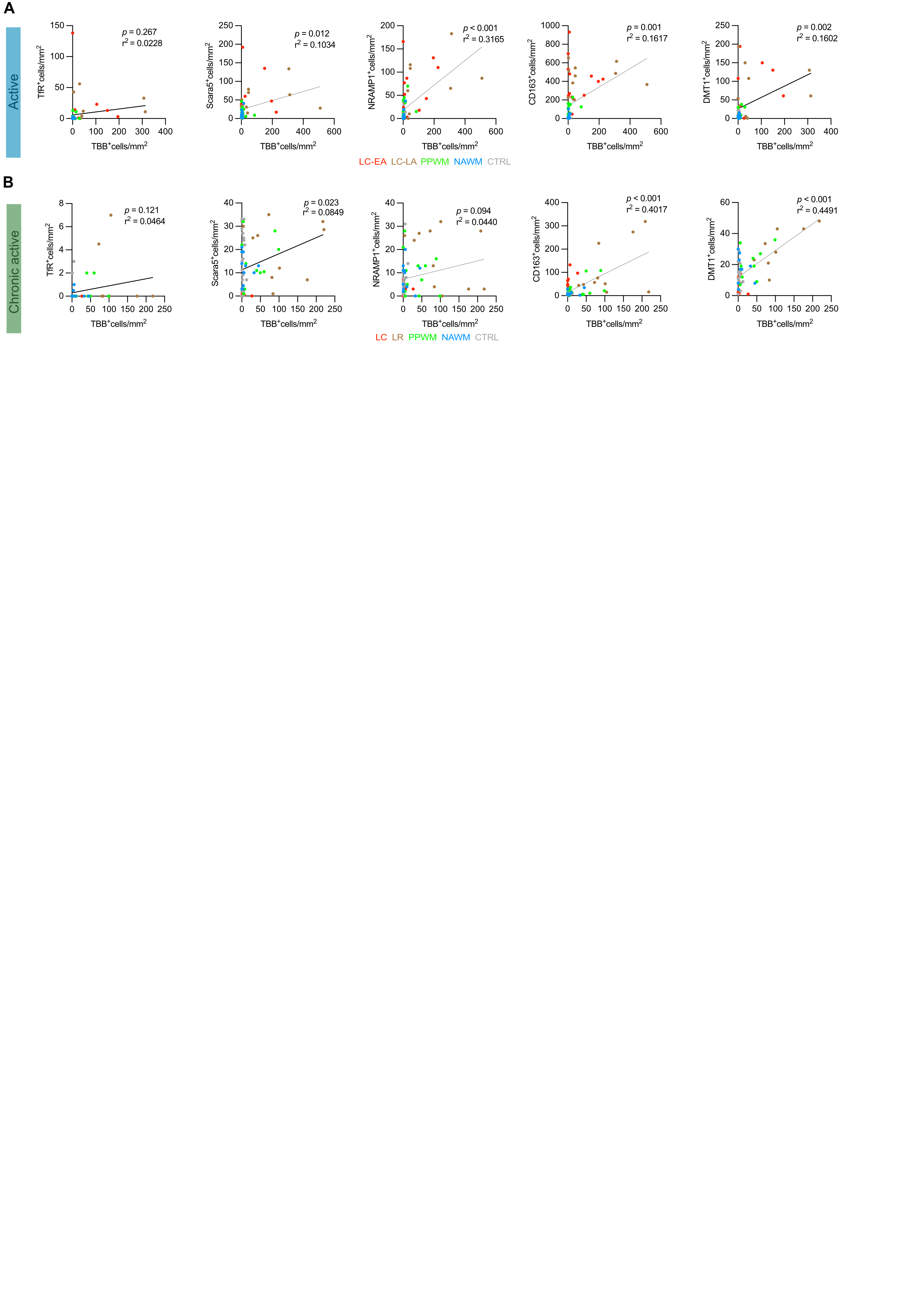

Supplement: Supplementary file 2 — Supplementary file2 (TIFF 953 KB) [file 401_2023_2627_MOESM2_ESM.tiff]

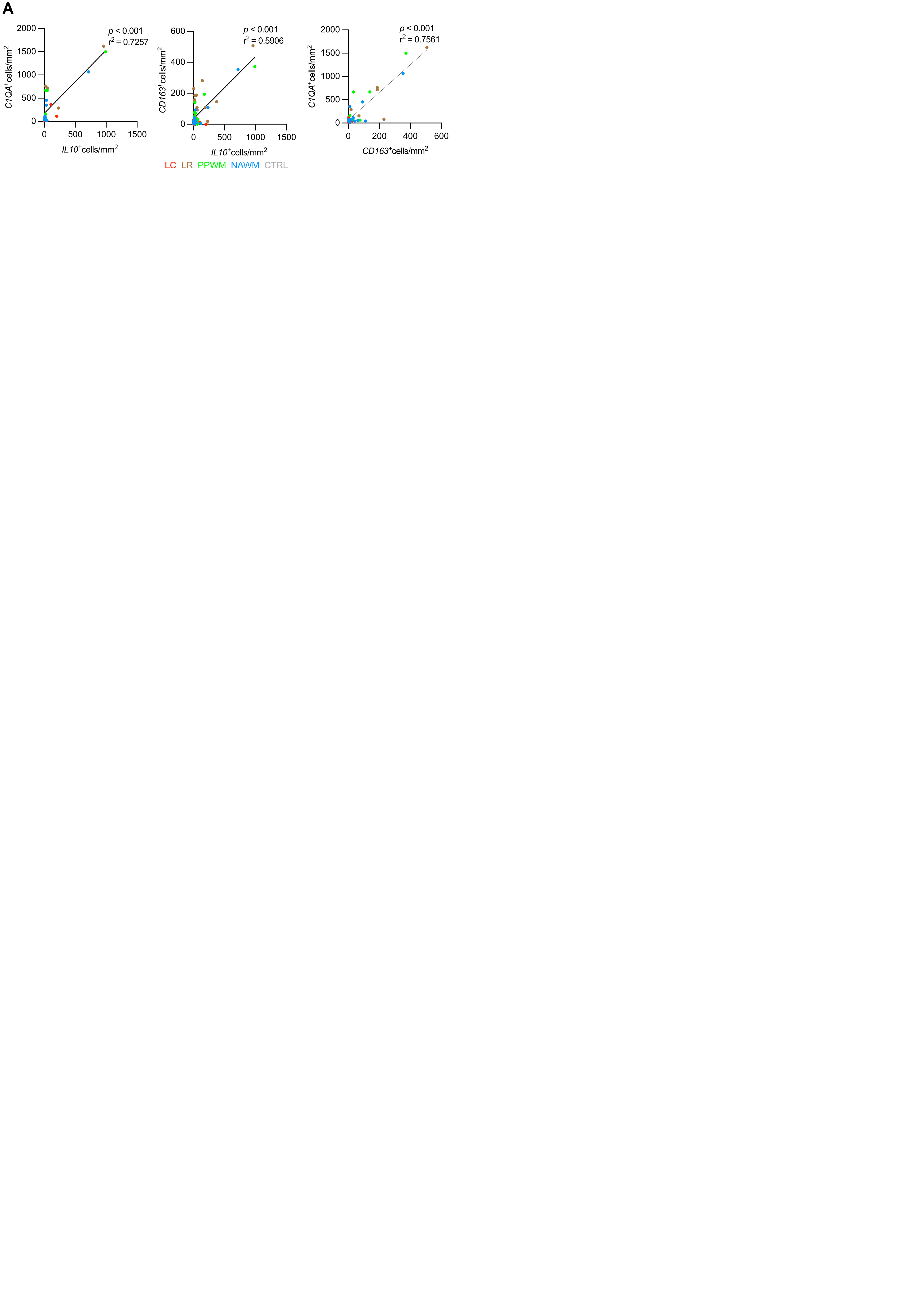

Supplement: Supplementary file 3 — Supplementary file3 (TIFF 473 KB) [file 401_2023_2627_MOESM3_ESM.tiff]
